# Effects of including of Japanese Pumpkin Seeds and Pomace in the Diets of Pacific White Shrimp (*Penaeus vannamei*)

**DOI:** 10.3390/ani13223480

**Published:** 2023-11-11

**Authors:** Thaise Dalferth Zancan, José María Monserrat, Robson Matheus Marreiro Gomes, Vilásia Guimarães Martins, Wilson Wasielesky, Marcelo Borges Tesser

**Affiliations:** 1 Institute of Oceanography, Federal University of Rio Grande, Av. Itália, km 8, Rio Grande 96203-900, Brazil; th4ise@gmail.com (T.D.Z.); roobinho_matheus@hotmail.com (R.M.M.G.); manow@mikrus.com.br (W.W.J.); 2 Laboratory of Aquatic Organisms Nutrition (LANOA), Rio Grande 96210-030, Brazil; 3 Institute of Biological Sciences, Federal University of Rio Grande, Av. Itália, km 8, Rio Grande 96203-900, Brazil; josemmonserrat@gmail.com; 4 Laboratory of Functional Biochemistry of Aquatic Organisms (BIFOA), Rio Grande 96210-030, Brazil; 5 School of Chemistry and Food, Federal University of Rio Grande, Av. Itália, km 8, Rio Grande 96203-900, Brazil; vilasiamartins@gmail.com; 6 Laboratory of Food Technology (LTA), Rio Grande 96203-900, Brazil; 7 Marine Shrimp Laboratory, Rio Grande 96210-030, Brazil

**Keywords:** *Cucurbita maxima*, *Cucurbita moschata*, pumpkin by-products, alternative ingredients, shrimp nutrition, phenolic compounds, antioxidant activity, carotenoids, color parameters

## Abstract

**Simple Summary:**

Pumpkin by-products such as seeds, and pomace can be a source of nutrients for aquatic organisms. In a study evaluating the inclusion of Japanese pumpkin seeds and pomace in the diet of the Pacific white shrimp *Penaeus vannamei*, we found that the seeds had a negative effect on shrimp performance and a pro-oxidative effect on muscle, whereas pumpkin pomace improved the feed conversion ratio and antioxidant activity of feeds and muscle and resulted in shrimp becoming more orange, both when fresh and after cooking. We do not recommend the inclusion of pumpkin seeds in the diets of *P. vannamei*, and we suggest further studies evaluating higher levels of inclusion of pumpkin pomace.

**Abstract:**

A 60-day feeding trial was conducted to evaluate the effects of including pumpkin seeds and pomace in the diets of Pacific white shrimp *Penaeus vannamei*, and the effects of these supplements on growth performance, body composition, and total polyphenol, flavonoid and carotenoid contents, as well as on total antioxidant activity, and body color parameters. Five diets were evaluated: pumpkin seeds (PS) at 50 and 100 g·kg^−1^, pumpkin pomace (PP) at 50 and 100 g·kg^−1^, and a control treatment. Pacific white shrimp (*P. vannamei*) juveniles (0.60 ± 0.01 g) were stocked in 15 tanks (310 L), containing 30 shrimps per tank, and the treatments were randomly distributed in triplicate. At the end of the experiment, shrimps were euthanized, weighed, and dissected for further analyses. The inclusion of PS in the diets impaired growth performance, reduced the total flavonoid content and had a pro-oxidative effect on muscle. The inclusion of PP in the diets did not affect growth performance, improved the feed conversion ratio, increased the total flavonoid content in the diets and hepatopancreas, and improved the antioxidant activity of the feeds and shrimp muscle. The total carotenoid content of the feeds increased with the inclusion of PS or PP in the diets; however, the total carotenoid content of shrimp increased only in those fed PP diets. Shrimp fed with PS diets showed a yellowish color and higher saturation when fresh and a reddish color and yellow hue angle after cooking. Shrimp fed PP diets turned reddish and yellowish, both when fresh and after cooking. The inclusion of PS in *P. vannamei* diets is not recommended; however, PP can be included at 100 g·kg^−1^ without affecting the growth parameters. Further studies evaluating the inclusion of higher PP levels in shrimp diets are recommended.

## 1. Introduction

Pumpkins are species of the genus *Cucurbita*, which belong to the Cucurbitaceae family and are represented by about 15 species, the main representatives being the species *Cucurbita maxima*, *Cucurbita moschata* and *Cucurbita pepo* [1,2]. Global pumpkin production exceeded 23.7 million tons in 2021, with China leading the global production, followed by India [3]. In Brazil, pumpkin production was 4.17 thousand tons in the last agricultural census of 2017, with production concentrated in the south and southeast regions [4]. The Japanese pumpkin (var. Tetsukabuto), popularly known as ‘Cabotiá’, is a hybrid species that crosses the *C. maxima* and *C. moschata* lineages [1,5]. This species has important cultivation characteristics, such as precocity, rusticity, high productive potential, quality, and long shelf life [1]. Pumpkin is mainly used in the production of jams and sweets. Therefore, large amounts of agro-industrial by-products are generated during industrial processing, consisting mainly of seeds, pomace, and husks, most of which are discarded [6].

Aquaculture is the fastest growing food production sector and has become very important in producing dietary protein sources and contributing to food security worldwide. Along with the increase in aquaculture production, the consumption of aquatic products has also increased [7]. Consequently, there is a growing demand for food to support the production of aquatic products intended for human consumption [8]. In recent years, with the emergence of the term “sustainability”, agro-industrial by-products have been successfully used to feed fish and shrimp [9,10]. The use of locally available agro-industrial by-products in aquaculture can be a way to conserve resources, reduce waste and operating costs, decrease dependency on fish and soybean meal, reduce waste disposal in the environment, develop new products and benefit these industries [9,10,11].

Pumpkin has attracted attention for being rich in vitamins and minerals [12,13,14,15,16]. Pumpkin by-products, such as leaves, flowers, seeds, and pomace, contain antioxidant compounds such polyphenols, and flavonoids known for their high bioactivity [12,13,14]. Pumpkin parts are important sources of minerals, phenolic compounds, flavonoids, and carotenoids [12]. Pumpkin seeds contain high concentrations of minerals such as potassium (K), calcium (Ca), manganese (Mn), phosphorus (P), and magnesium (Mg) [15,16]. Phenolic compounds such as p-hydroxybenzoic acid, caffeic acid, ferulic acid, and vanillic acid are found in pumpkin seeds [14,17]. In addition to p-hydroxybenzoic acid, pumpkin pomace contains coumaric acid, rutin, chlorogenic acid, syringic acid, cinnamic acid, and quercetin [18]. These bioactive compounds inhibit the production of free radicals and reactive oxygen species, protect health, and reduce the risk of serious pathologies [12,19]. Pumpkin seeds and pomace are rich in carotenoids, particularly β-carotene, a provitamin A carotenoid [20,21]. The high concentration of carotenoid in pumpkin can improve the color parameters of aquatic organisms, which is of great importance in the consumer market [22]. Pumpkin may contain some small amounts of antinutritional factors associated with reduced absorption of macronutrients such as phytic acid, oxalate, and some toxins such as cucurbitin, cucurmosin and cucurbitacin; however, the quantities of these antinutritional factors in pumpkin are low [13].

Pacific white shrimp (*Penaeus vannamei*) is the crustacean species with the highest production in aquaculture, representing almost 52% of the total production [23]. Several studies have explored the potential use of agro-industrial by-products as alternative ingredients for the nutrition of *P. vannamei*, such as grape bagasse [24], peanut meal [25] and fermented cottonseed meal [26]. However, there are few studies related to the inclusion of pumpkin by-products in the nutrition of aquatic organisms. From a nutritional perspective, pumpkin seeds can help improve the growth performance and feed efficiency of fish [27]. There are no data on the inclusion of pumpkin by-products in shrimp nutrition. Thus, this study aimed to evaluate the effects of the inclusion of Japanese pumpkin seeds and pomace on the nutrition of Pacific white shrimp *P. vannamei* and the effects of these on growth performance, body composition, phenolic compounds, antioxidant activity, total carotenoid content, and shrimp body color parameters.

## 2. Materials and Methods

### 2.1. Location and Facilities

The study was conducted at the Marine Station of Aquaculture (EMA) of the Institute of Oceanography (IO) of the Federal University of Rio Grande (FURG), located at Cassino Beach, Rio Grande, RS, Brazil.

### 2.2. Preparation and Analyses of the Pumpkin By-Products

The Japanese pumpkin by-products were obtained through a donation from Massas Nona Oliva (Vacaria, RS, Brazil). The seeds were manually separated from the pomace, and then dried at 60 °C for 24 h in an air recirculating oven (MA 035, Marconi, Piracicaba, Brazil), ground in a rotor mill (MA-090, Marconi, Piracicaba, Brazil) and sieved (mesh 28) to obtain the powder. Analyses of the proximal composition of Japanese pumpkin seeds and pomace powders were carried out at the Laboratory of Aquatic Organisms Nutrition (Laboratório de Nutrição de Organismos Aquáticos—LANOA) of the Federal University of Rio Grande (FURG). Moisture, ash, crude protein, crude fiber [28] and total lipid [29] contents are described in Table 1. Nitrogen-free extracts (NFE) were calculated based on nutrient analyses using the following equation:

(1) NFE(%)=100−(ash+crude protein+crude fiber+total lipid content)

Analyses of total polyphenol [30] and carotenoid contents [31,32] was carried out at the Laboratory of Functional Biochemistry of Aquatic Organisms (Laboratório de Bioquímica Funcional de Organismos Aquáticos—BIFOA) (FURG) and are also reported in Table 1.

### 2.3. Experimental Diets

Five iso-nitrogenous (340 g·kg^−1^ crude protein) and iso-lipidic (90 g·kg^−1^ total lipid content) experimental diets were formulated. In this study, two diets were formulated with seeds with peel, including 50 g·kg^−1^ (PS50) and 100 g·kg^−1^ of pumpkin seeds powder (PS100). Two diets were formulated with pomace, including 50 g·kg^−1^ (PP50) and 100 g·kg^−1^ of pumpkin pomace powder (PP100), and a control diet was formulated without the inclusion of pumpkin seeds or pomace powder. The inclusion levels of Japanese pumpkin by-products were based on previous studies that evaluated the inclusion of pumpkin seeds in fish feed [22] and the inclusion of pumpkin pomace in monogastric feeds, such as pigs [33] and broilers [34]. The formulations and nutrient profiles of the experimental diets are presented in Table 2. During diet preparation, all the dry ingredients were weighed and manually homogenized, and fish oil and water were added. Pellets were produced using a meat grinder, and then dried at 60 °C in an air-recirculation oven (Marconi, MA 035) for 24 h and stored in a freezer (−18 °C) until further use.

### 2.4. Experimental Design

Pacific white shrimp (*P. vannamei*) juveniles nursed in the Marine Shrimp Laboratory (Laboratório de Carcinocultura—EMA/FURG) with an average weight of 0.60 ± 0.01 g were transferred to 15 circular polyethylene tanks with a volume of 310 L each, arranged in five treatments in triplicate containing 30 shrimps per tank. The clear-water system had constant aeration. In a 60-day feeding trial, shrimp were fed twice a day (8:00 and 16:00 h), with a feeding rate according to Jory et al. [35]. For this trial, shrimp growth was monitored weekly using partial biometry, with 10 shrimp being weighed per tank and the feeding rate determined based on average weight.

### 2.5. Water Quality Parameters

During the experimental period, dissolved oxygen and temperature were measured daily before feeding through a digital multi-parameter oximeter (YSI 200A, Tecnal, Piracicaba, Brazil), and pH was determined with a digital pH meter (Seven2Go, Mettler Toledo, Barueri, Brazil). Total ammonia (TAN − NH_3_ + NH_4_^+^) was determined following the methodology of UNESCO [36] and nitrite (N − NO_2_^−^) following the methodology of Aminot and Chaussepied [37]. Measurements of alkalinity [38] and salinity (Alfakit, portable refractometer) were performed weekly. The tanks were siphoned daily to remove feces and remaining feed. Approximately 80% of the total volume of each tank was exchanged when the total ammonia concentrations exceeded 1 mg TAN·L^−1^.

### 2.6. Data Collection and Assessed Variables

At the end of the experiment, all shrimp were fasted for 24 h, euthanized on ice and weighed to assess growth performance. The evaluated growth parameters were weight gain (WG), protein efficiency ratio (PER), feed conversion ratio (FCR), survival, specific growth ratio (SGR) and relative weight gain (RWG), and were calculated according to the following equations:

(2) $$WG=Final\;weight\;\left(g\right)-Initial\;weight\;(g)$$

(3) $$PER=\frac{Weight\;gain\;(g)}{Feed\;intake\;\left(g\right)\times crude\;protein\;(\%)}\times 100$$

(4) $$FCR=\frac{Feed\;intake\;(g)}{Weight\;gain\;(g)}$$

(5) $$Survival=\frac{Final\;number\;of\;shrimp}{Initial\;number\;of\;shrimp}\times 100$$

(6) $$SGR=\frac{ln\;(final\;weight)-ln\;(initial\;weight)}{Days\;of\;feeding}\times 100$$

(7) $$RWG=\frac{Final\;weight-initial\;weight}{Initial\;weight}\times 100$$

For determination of total polyphenol and flavonoid contents, and total antioxidant activity, three shrimps from each tank were dissected for organ collection (hepatopancreas and muscle) and the samples were stored at −80 °C. For the analysis of shrimp body color, six shrimp from each tank were randomly selected and euthanized immediately. The analysis was performed using three fresh shrimps and three that were cooked immediately before the analysis. For the whole-body proximal composition analysis, nine shrimp from each tank were euthanized on ice and stored at −18 °C until the analysis was carried out.

### 2.7. Whole-Body Proximate Composition

The whole-body proximate composition of the shrimp samples was analyzed at the Laboratory of Aquatic Organisms Nutrition (LANOA/FURG). The moisture content was calculated from the weight of the samples before and after drying in a drying oven (Q317M-52, Quimis, Diadema, Brazil) at 105 °C for 12 h [28]. The ash content was determined gravimetrically after burning in a muffle furnace (MA 385, Marconi, Piracicaba, Brazil) at 600 °C for 5 h [28]. Total nitrogen was determined by the micro-Kjeldahl method (MA-036, Marconi, Piracicaba, Brazil), and crude protein content was calculated as N × 6.25 [28]. The total lipid content was measured using the cold extraction method [29].

### 2.8. Biochemical Analyses

Biochemical analyses were performed at the Laboratory of Functional Biochemistry of Aquatic Organisms (BIFOA/FURG). The hepatopancreas and muscle were weighed and homogenized in methanol at a 1:2 ratio (*w*/*v*), whereas the feeds were homogenized at a 1:3 ratio (*w*/*v*). The samples were sonicated (Q55 Sonicator, QSonica, Newtown, Brazil) at 50 V for 60 s, then stirred (KLA-210, Satra, Araucária, Brazil) under refrigeration at 80 rpm for 3 h and centrifuged at 9.940 rpm and 4 °C for 30 min to collect the supernatants. The methanolic extracts were stored at −80 °C and used to determine the total polyphenol [30] and flavonoid [39] contents, and antioxidant activity. 

#### 2.8.1. Determination of Total Polyphenol and Flavonoid Contents

Polyphenol content was measured using the Folin–Ciocalteu method described by Waterhouse [30]. Folin–Ciocalteu reagent (100 µL) and distilled water (1.58 mL) were added to 20 µL of the sample. This blend was allowed to stand for 8 min before adding 300 µL of sodium carbonate (Na_2_CO_3_) 25% solution. The solution was allowed to stand for 2 h before measurement at 765 nm. Data are expressed in µg of gallic acid equivalent (GAE) per gram of crude extract, based on the gallic acid calibration curve. 

Flavonoid content was determined by reaction with aluminum chloride (AlCl_3_) using the method described by Gajula et al. [39]. Distilled water (50 µL) and sodium nitrite (NaNO_2_) 5% solution (37.5 µL) were added to 50 µL of the sample. This blend was allowed to stand for 6 min before adding 75 µL AlCl_3_ 10% solution and another 5 min before adding sodium hydroxide (NaOH) 1M solution (250 µL). The solution was allowed to stand for 30 min before measuring absorbance at 510 nm. Data are expressed as µg of quercetin equivalent (QE) per gram of crude extract, based on the quercetin calibration curve.

#### 2.8.2. Determination of Total Antioxidant Activity

Antioxidant activity was determined using the 2,2-diphenyl-1picrylhydrazyl (DPPH) radical scavenging method [40]. A DPPH 60 µM solution was prepared and 240 µL was added to 40 µL of muscle samples, 1.2 mL to 20 µL of hepatopancreas samples and 2.0 mL to 20 µL of feed samples. The samples were immediately read at 515 nm, at 1 min intervals for 15 min. The total antioxidant activity was determined using the equation:

(8) $$Antioxidant\;activity\left(\%\right)=\frac{\left({Abs}_{b}-{Abs}_{s}\right)}{{Abs}_{b}}\times 100$$

where Abs_b_ is the absorbance of the blank and Abs_s_ is the absorbance of the sample.

#### 2.8.3. Determination of Total Carotenoid Content

Total carotenoid content (TCC) was determined according to Yanar et al. [31], with modifications suggested by Rosas et al. [32]. Samples of 0.25 g of the feeds and the total dry body samples were tested in duplicate. Anhydrous sodium sulfate was added to the samples at the same proportion (0.25 g) and then they were macerated. The mixture was washed with acetone (5 mL), transferred to an amber glass bottle and left to stand for three days in the dark at room temperature. The samples were then centrifuged at 6510 rpm for 5 min. The absorbance of the supernatants was measured at 480 nm. The total carotenoid content was determined using the equation:

(9) $$TCC\left({µg\cdot g}^{-1}\right)=\frac{Abs\times K\times V}{E\times G},$$

where Abs is the absorbance at 480 nm, K is a constant (104), V is the volume extracted from the solution (ml), E is the extinction coefficient (1900 k), and G is the weight of the sample (g).

### 2.9. Color Parameters of Shrimp

For each treatment, nine fresh shrimps and nine cooked shrimps were evaluated using a digital chromometer (CR 400, Minolta, Japan) that measures the light reflectance of the samples. The color parameters used were lightness (L*), green/red chromaticity (a*) and blue/yellow chromaticity (b*). The chromometer was placed on the abdominal region of the shrimp, and measurements were performed. Chroma (C_ab_) and hue angle (H_ab_) values were calculated from the values of parameters a* and b*, according to Hunt [41], using the equations:

(10) Cab=(a*2+b*2)12,

(11) $$H_{ab}={tan}^{-1}\left(\frac{b*}{a*}\right),\;\mathrm{when}\;a*>0$$

(12) $$H_{a*b}=180+{tan}^{-1}\left(\frac{b*}{a*}\right),\;\mathrm{when}\;a*\leq 0$$

Chroma refers to color saturation (higher values indicate more intense color perception). Hue angle (0° = red hue; 90° = yellow hue; 180° = green hue) expresses the relation between the colors.

### 2.10. Statistical Analyses

All data are expressed as the mean ± standard deviation. The homogeneity and normality of the data were assessed using the Bartlett and the Shapiro–Wilk tests, respectively. A one-way analysis of variance (ANOVA) was used to compare data among treatments, and when statistical differences between treatments were found, the means were compared according to Tukey’s test, with a significance level of 5% (*p* < 0.05).

## 3. Results

### 3.1. Water Quality Parameters

The water quality parameters are listed in Table 3. No statistically significant differences (*p* > 0.05) were detected among treatments of the evaluated parameters.

### 3.2. Growth Performance

The growth performance of shrimp fed diets containing Japanese pumpkin seeds (Table 4) showed significant differences (*p* < 0.05) in the final weight (FW), weight gain (WG), feed conversion ratio (FCR), specific growth ratio (SGR), and relative weight gain (RWG) at the end of the trial period. In the pumpkin seed diets, the control treatment was more efficient for FW, WG, FCR, SGR, and RWG, indicating that pumpkin seeds are not a viable ingredient for improving shrimp growth parameters.

In treatments with pumpkin pomace, shrimp from the PP50 treatment showed a better FCR (1.82 ± 0.12). Shrimp from PP50 and PP100 also showed a better PER than those fed with the control diet (*p* < 0.05). No statistical differences (*p* > 0.05) were observed in the other evaluated performance parameters.

### 3.3. Whole-Body Proximal Composition

In regard to the whole-body proximal composition of shrimp, there were significant differences (*p* < 0.05) only for ash in the PS treatments, where increasing pumpkin seed inclusion levels increased the body ash content (Table 5).

### 3.4. Total Polyphenol and Flavonoid Contents

**Figure 1:**
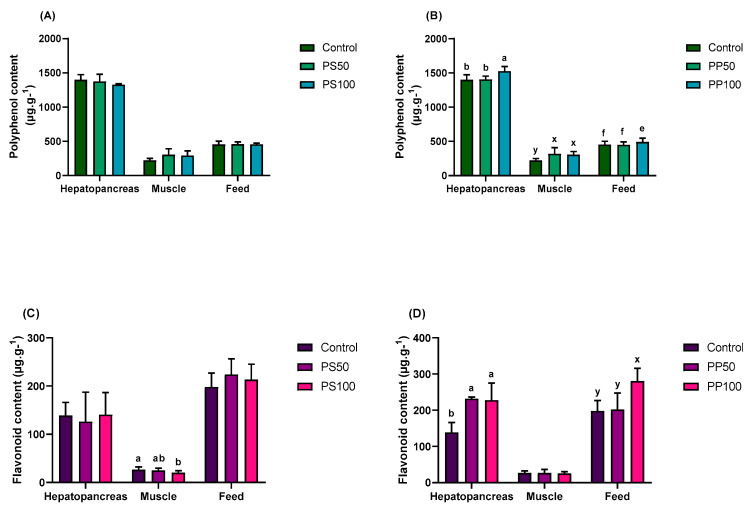
Total polyphenol and flavonoid content of the tissues and feeds with the inclusion of Japanese pumpkin seeds (**A**,**C**) and pomace (**B**,**D**). The bars indicate the mean ± standard deviation (*n* = 9). Values with different letters have significant differences according to Tukey’s test (*p* < 0.05).

As shown in Figure 1A, the total polyphenol content did not differ (*p* > 0.05) among the groups fed PS diets and the control treatment, either in the tissues or feed. Meanwhile, in the groups fed PP diets, the inclusion of pumpkin pomace increased the levels of total polyphenols in the hepatopancreas, muscle and in the feeds (*p* < 0.05; Figure 1B). The hepatopancreas showed a higher content of polyphenols in the PP100 diet (1525.85 ± 68.37 µg·g^−1^). Levels in the muscle increased both in the PP50 diet (319.18 ± 90.48 µg·g^−1^) and in the PP100 diet (306.06 ± 47.15 µg·g^−1^) compared with the control (222.57 ± 28.55 µg·g^−1^). These results indicate an increase in polyphenols in the tissues of shrimp fed diets containing pumpkin pomace, with the PP100 feed having the highest polyphenol content (492.59 ± 54.93 µg·g^−1^) (Figure 1B).

As shown in Figure 1C, the total flavonoid content did not differ among pumpkin seed feeds (*p* > 0.05). However, the PS diets decreased total muscle flavonoid content (26.51 ± 5.49, 24.78 ± 5.07 and 20.11 ± 4.24 µg·g^−1^). The hepatopancreas showed no differences in the total flavonoid content among the PS diets (*p* > 0.05). Unlike the PS diets, there were no differences in the total flavonoid content in muscle among the PP diets (*p* > 0.05; Figure 1D). However, diets with a 100 g·g^−1^ inclusion of pumpkin pomace showed a higher total flavonoid content (280.48 ± 35.34 µg·g^−1^), and it also increased in the hepatopancreas of shrimp fed the PP diets (138.99 ± 27.24, 231.77 ± 4.76 and 227.69 ± 47.57 µg·g^−1^ in control, PP50 and PP100, respectively; Figure 1D).

### 3.5. Total Antioxidant Activity

**Figure 2:**
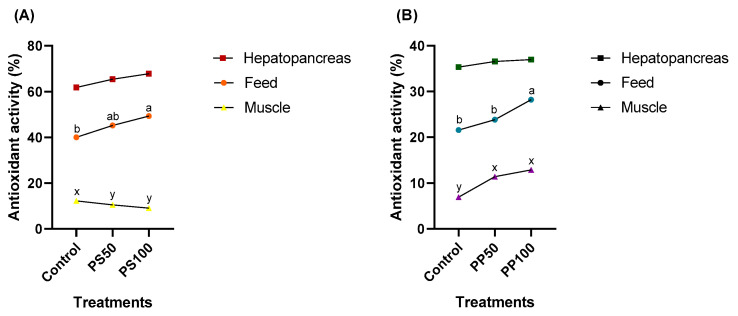
Total antioxidant activity of the shrimp tissues and feeds with the inclusion of Japanese pumpkin seeds (**A**) and pomace (**B**). The data points represent the mean ± standard deviation (*n* = 9). Values with different letters have significant differences according to Tukey’s test (*p* < 0.05).

As shown in Figure 2, the total antioxidant activity in the hepatopancreas remained the same for both pumpkin seeds (Figure 2A) and pumpkin pomace diets (Figure 2B) (*p* > 0.05). Greater antioxidant activity was observed in feeds PS50 and PS100 (45.23 and 49.31%, respectively). However, despite the increased antioxidant activity of diets with pumpkin seeds, the muscle showed reduced antioxidant activity in shrimp fed PS50 and PS100 (10.53 and 9.13%, respectively) (Figure 2A).

The opposite trend was observed for the PP treatments (Figure 2B). The PP100 feed increased total antioxidant activity (54.02%). Furthermore, as the inclusion of pumpkin pomace in the diets increased, the muscle showed an increase in the total antioxidant activity (16.80 and 16.59%, respectively) (Figure 2B).

### 3.6. Total Carotenoid Content (TCC)

**Figure 3:**
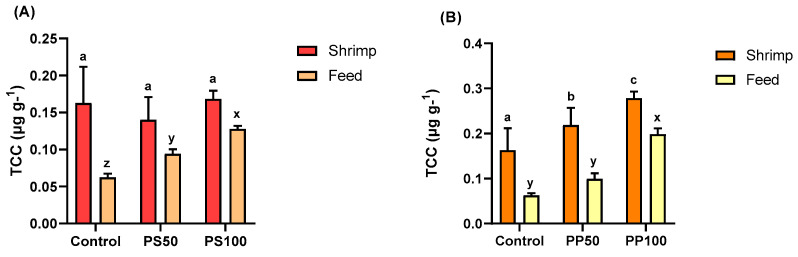
Total carotenoid content (TCC) of the feeds and shrimps fed diets with the inclusion of Japanese pumpkin seeds (**A**) and pomace (**B**). Bars represent the mean ± standard deviation (*n* = 9). Values with different letters have significant differences according to Tukey’s test (*p* < 0.05).

As shown in Figure 3, due to the high levels of carotenoid found in the Japanese pumpkin seeds (0.72 ± 0.03 µg·g^−1^) and pomace (1.25 ± 0.04 µg·g^−1^), the total carotenoid content was higher in both the pumpkin seeds (Figure 3A) and pumpkin pomace (Figure 3B) feeds (*p* < 0.05). There were no significant differences in the total carotenoid content in the shrimps fed PS diets (*p* > 0.05) (Figure 3A). However, the total carotenoid content increased in shrimp fed the PP diets as the proportion of pumpkin pomace in the diet increased (0.162 ± 0.04 to 0.219 ± 0.03 and 0.278 ± 0.01 µg·g^−1^, in control, PP50 and PP100, respectively) (Figure 3B).

### 3.7. Color Parameters

**Figure 4:**
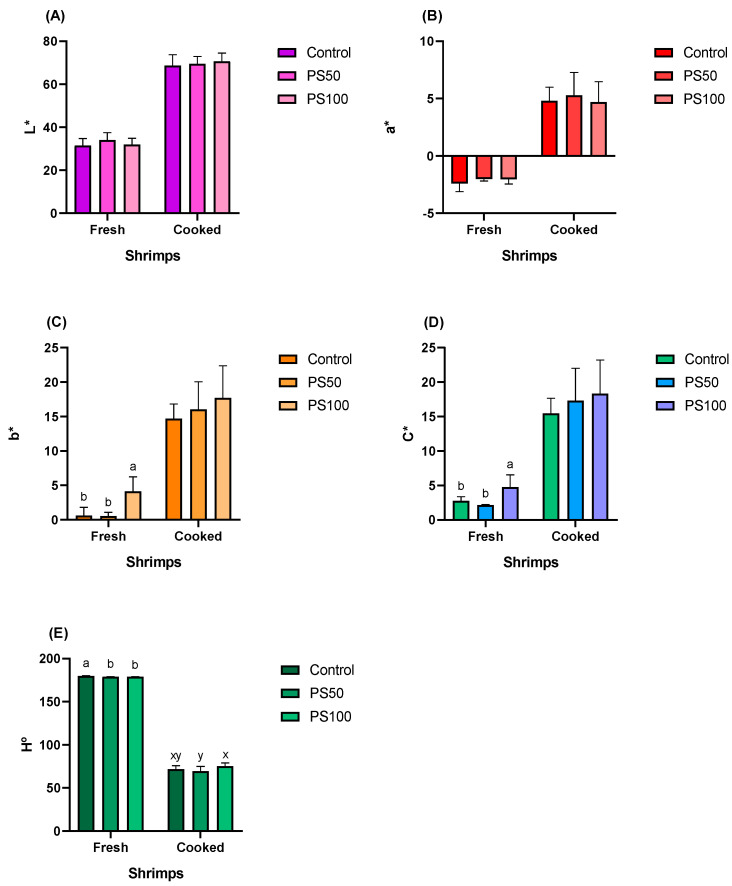
Color parameters of fresh and cooked shrimps fed diets with the inclusion of Japanese pumpkin seeds. (**A**) lightness (L*) ranges between 0 (black) and 100 (white); (**B**) the a* parameter ranges from negative (green) to positive values (red); (**C**) the b* parameter ranges from negative (blue) to positive values (yellow); (**D**) chroma (C*) measures the saturation of the color; (**E**) hue angle (H°) is the ratio between redness and yellowness. Bars represent the mean ± standard deviation (*n* = 9). Values with different letters have significant differences according to Tukey’s test (*p* < 0.05).

As shown in Figure 4, in the treatments evaluating the inclusion of Japanese pumpkin seeds in the diets of shrimp, there were no significant differences (*p* > 0.05) in the parameter of lightness (L*) (Figure 4A) and a* (Figure 4B), either in fresh or cooked shrimps. A yellowish color (b*) (Figure 4C) and greater saturation (C*) (Figure 4D) were observed in fresh shrimp fed with PS100 (*p* < 0.05). The hue angle (H°) became yellowish in shrimp fed with diets that include pumpkin seeds, both for fresh and cooked shrimps (Figure 4E).

**Figure 5:**
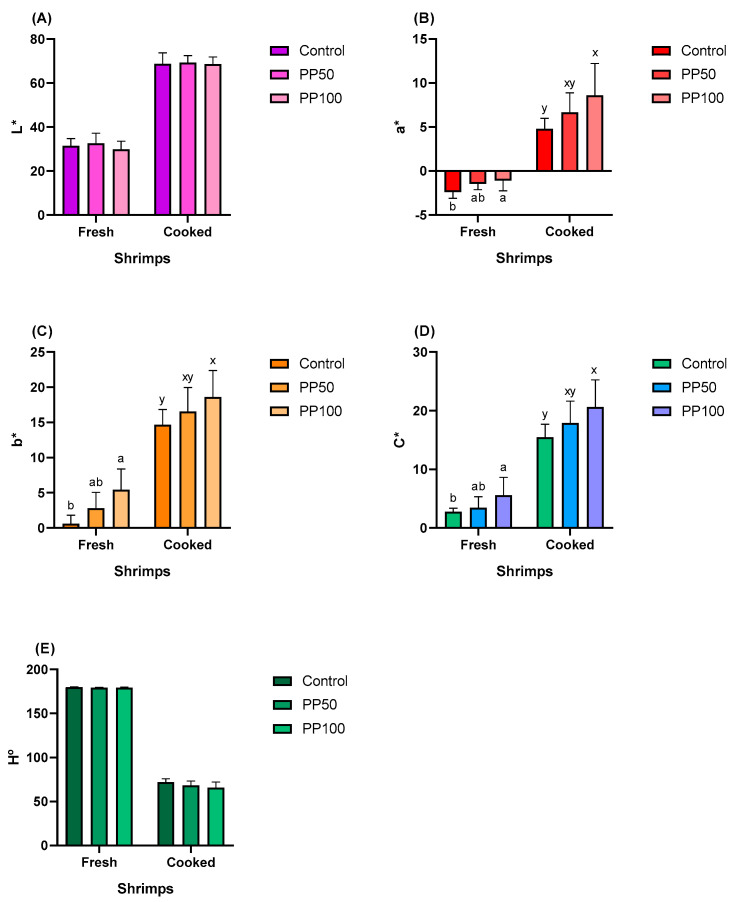
Color parameters of fresh and cooked shrimps fed diets with the inclusion of Japanese pumpkin pomace. (**A**) lightness (L*) ranges between 0 (black) and 100 (white); (**B**) the a* parameter ranges from negative (green) to positive values (red); (**C**) the b* parameter ranges from negative (blue) to positive values (yellow); (**D**) chroma (C*) measures the saturation of the color; (**E**) hue angle (H°) is the ratio between redness and yellowness. Bars represent the mean ± standard deviation (*n* = 9). Values with different letters have significant differences according to Tukey’s test (*p* < 0.05).

As shown in Figure 5, in the treatments evaluating the inclusion of Japanese pumpkin pomace, there was no difference (*p* > 0.05) in lightness (L*), either in fresh or cooked shrimps (Figure 5A). Reddish (a*) (Figure 5B), yellowish colors (b*) (Figure 5C) and saturation (C*) (Figure 5D) were more evident when shrimp were fed PP100, in both fresh and cooked shrimp (*p* < 0.05). No differences in hue angle (H°) were observed among the treatments (*p* > 0.05) (Figure 5E). The increased coloration in shrimp fed the highest inclusions of Japanese pumpkin seeds (PS100) and pomace (PP100) may be related to the higher concentration of total carotenoids in these diets, as well as in the body of the shrimp.

## 4. Discussion

Few studies have been conducted on the inclusion of pumpkin by-products in the diet of aquatic organisms, and most of these studies have evaluated the use of seeds. Pumpkin seeds are used in fish feed mainly because of their high protein content and unsaturated fatty acids concentration [22,27]. The results obtained for the proximal composition and total polyphenols and carotenoid contents of Japanese pumpkin seeds and pomace indicate the quality of these by-products, which prompted us to evaluate their inclusion in shrimp *P. vannamei* feeds.

The water quality parameters of this 60-day feeding trial of *P. vannamei* fed diets with Japanese pumpkin seeds and pomace were within the optimum range [42], and therefore, could not have interfered with growth parameters. Our findings showed a reduction in the growth parameters of shrimp fed pumpkin seeds diets. The final weight, weight gain, feed conversion ratio, specific growth ratio and relative weight gain were affected by the inclusion of pumpkin seeds in both treatments. Our results agree with those of Lovatto et al. [43], who found that the inclusion of pumpkin seeds in the diet of silver catfish (*Rhamdia quelen*) reduced fish growth ratios. Murray et al. [44] also showed that partial replacement of fishmeal and fish oil with pumpkin kernel cake and rapeseed oil led to a reduction in growth ratios and decreased biomass of *Salvelinus alpinus* juveniles, particularly at the higher dietary intake ratio. In our study, there were no differences in FCR among the PS diets. Greiling et al. [22] did not find differences in FCR in rainbow trout and brook trout fed pumpkin seed cake diets and the reference diet, but there was a tendency for better FCR in the pumpkin seed cake diets. In our study, the decrease in growth parameters could be attributed to the higher crude fiber content in the PS diets. Diets with the inclusion of 50 and 100 g·kg^−1^ of Japanese pumpkin seeds had an increase of 116.55 and 259.31% in crude fiber, respectively, compared with the control diet. Furthermore, with the increased inclusion of pumpkin seeds in the diets, there was a decrease in corn starch and soybean meal, which are better utilized by shrimp [45]. 

To our knowledge, pumpkin pomace has been used as a feed ingredient in some species of monogastrics, such as pigs [33] and broilers [34]. In our study, the growth parameters and survival of shrimp fed diets including Japanese pumpkin pomace were not affected. Furthermore, the feed conversion and protein efficiency ratios were better in treatments with Japanese pumpkin pomace inclusion. Pumpkin pulp is rich in starch and soluble fiber, mainly pectin, which are responsible for improving digestibility [12,46]. Pectin can act as a natural prebiotic, stimulating antioxidant defenses and improving the immune system of aquatic organisms [47,48]. Owing to these properties, pectin can also act as a growth promoter in fish, considering that it influences cellular activity and regulates the intestinal microbiota [49]. Moreover, the higher concentration of polyphenols and flavonoids in PP diets may also have contributed to the improved FCR values. This was the case, for example, for *P. monodon* fed on diets rich in polyphenols extracted from sugarcane [50].

In our study, the whole-body proximal composition of shrimp was not affected, except for ash content in shrimp on PS diets (*p* < 0.05), indicating higher mineral deposition in shrimp muscle. Pumpkin seeds are high in magnesium, potassium, and phosphorus, as well as other minerals such as zinc, manganese, iron, calcium, sodium, and copper [15].

Phenolic compounds such as phenolic acids and flavonoids, are the main secondary compounds produced by plants and are composed of two or more benzene rings, each having at least one hydroxyl group. These compounds perform several functions such as growth, pigmentation, reproduction, protection against ultraviolet radiation and resistance to pathogens and herbivores [51]. Phenolic compounds act as antioxidants because of the reactivity of their phenolic moiety, and their antioxidant activity predominantly consists of scavenging free radicals through the donation of hydrogen atoms. In addition, they contribute to the organoleptic properties of leaves, flowers, and fruits, such as bitterness, astringency, color, and flavor [17,51,52,53]. Pumpkin seeds and pomace have a high content of phenolic compounds. The main phenolic compounds identified in pumpkin seeds are p-hydroxybenzoic, caffeic, ferulic, and vanillic acids [17]. In pomace, in addition to p-hydroxybenzoic acid, the main phenolic compounds are coumaric acid and rutin, followed by chlorogenic acid, syringic acid, cinnamic acid, and quercetin [18]. In our study, the inclusion of pumpkin seeds in diets did not influence the bioaccumulation of polyphenols in shrimp tissues or in the feed. However, with the inclusion of pumpkin pomace in the diets, there was a greater bioaccumulation of polyphenols in the hepatopancreas (PP100), in the muscle (PP50 and PP100) and in the feed (PP100). The bioavailability of polyphenols refers to the fraction released from the food matrix, digested, absorbed, metabolized in the liver and intestine, and subsequently released to target tissues and cells, where they exert their bioactivity [51]. This may have occurred because of the pumpkin pomace having a higher total polyphenol content (1650.00 ± 85.85 µg·g^−1^) than the seeds (685.43 ± 34.92 µg·g^−1^). Furthermore, the PS50 and PS100 diet formulations resulted in a larger reduction (−8.29–−16.55%, respectively) in soybean meal than the PP diets (−5.42–−10.81%, respectively) compared with the control diet. Soybean meal is rich in phenolic compounds, mainly isoflavones and saponins [54]. As a result, the increase in the total polyphenol content of the diet with higher inclusion of pumpkin pomace may also be related to the smaller reduction of soybean meal in the formulation.

Similar to the total polyphenol content results, the inclusion of pumpkin seeds in the diets did not change the flavonoid content among the diets or in the shrimp hepatopancreas. However, total muscle flavonoid content decreased with increasing seed inclusion in the diets. Some antioxidant compounds, such as flavonoids and tocopherols, that react with molecular oxygen are reducing agents and can act as pro-oxidants when a reduced metal or transition metals are available [54,55] Furthermore, some phenolic compounds have little or no biological activity in vivo, leading to a low bioavailability in the organism [56]. Unlike treatments with pumpkin seeds, the addition of pumpkin pomace increased the total flavonoid content in the diets and hepatopancreas, resulting in higher values than those observed in the diets with seeds. Flavonoids, such as quercetin, found in pumpkin pomace are important modulators of the intestinal microbiota, promoting prebiotic effects, improved growth ratios, antioxidant effects and protection against pathogenic microorganisms [57,58]. 

Antioxidant activity is important for reducing oxidative stress, increasing free radical scavenging, and promoting health and immunity in aquatic organisms. Pumpkin seeds have a high antioxidant activity [59], and this activity is due to the presence of phenolic compounds in the pumpkin, as previously discussed. In our study, the inclusion of pumpkin seeds in the shrimp diet increased the antioxidant activity of the diets. However, the antioxidant activity was slightly lower in the muscle, probably because of the lower accumulation of flavonoids in this tissue, which was also reflected in the reduced growth parameters of shrimp fed the PS diets. In the diets containing pumpkin pomace, there was an increase in the antioxidant activity of the feed and in the shrimp muscle. This increase in the antioxidant capacity of diets containing pumpkin pomace and in the muscle of shrimp fed these diets may be related to the quantity of total polyphenols and flavonoids, as well as carotenoids. Carotenoids play an important role in scavenging free radicals [59]. Abbas et al. [60] detected an increase in the antioxidant activity of pumpkin *C. maxima*, correlating it with high levels of carotenoids such as neoxanthine, violanthin, lutein and β-carotene.

Color is an important indicator of crustacean quality and has a direct impact on consumer acceptability. Consumers prefer to buy shrimp that are light grey in color and turn an intense orange color when cooked [61]. The color of shrimp is due to the accumulation of crustacyanin, which is formed by a protein–astaxanthin complex [62]. During cooking, color changes in crustaceans are closely related to the dissociation of astaxanthin (3,3′-dihydroxy-β,β’-carotene-4,4′-dione) from its pigment proteins [63]. Astaxanthin is the major fat-soluble pigment carotenoid found in a variety of aquatic animals and is responsible for imparting a wide variety of colors to these animals [64,65]. In marine invertebrates, β-carotene is converted to astaxanthin, changing in color from yellow to red, blue, or purple according to the need for camouflage of different species [66]. However, shrimp and other crustaceans are unable to biosynthesize carotenoids in the body, and require a diet supplemented with carotenoid sources [66]. Astaxanthin is the most important and expensive carotenoid used in aquaculture for pigmentation of salmon, trout, and shrimp [67]. Thus, several studies have evaluated dietary supplementation of shrimp with natural sources of carotenoids [68,69,70]. Carotenoids are a group of isoprenoid metabolites synthesized by photosynthetic organisms such as plants, algae, and cyanobacteria. In plants, they are essential pigment compounds in photosynthesis and light-protection processes [71]. The main carotenoids found in pumpkin are lutein and β-carotene, which are responsible for the varied colors of yellow, orange, and red found in the fruits, seeds, and peels of pumpkin. Lutein is mostly found in pumpkin seed oil, whereas β-carotene is found in significant amounts in pulp and peel [21]. According to our findings, the inclusion of pumpkin seeds and pomace increased the concentration of total carotenoids in the diet, with the highest levels obtained with pumpkin pomace. Shrimp fed diets containing pumpkin seeds did not differ from the control treatment in terms of total carotenoid content. However, in shrimp fed diets containing pumpkin pomace, the total carotenoid content increased with the inclusion of pumpkin pomace. We observed that the bioavailability of carotenoids was higher in the PP diet than in the PS diet. The bioavailability of carotenoids can be affected by dietary factors such as the nature of the carotenoids, the lipid content of the diet, and the interaction between carotenoids and other dietary components, especially fiber, which affect the viscosity of gastrointestinal contents, the bioavailability of salts in bile ducts, and enzymatic lipolysis of triglycerides [72]. The improvement in shrimp color was associated with the total carotenoid content in shrimp feed containing pumpkin seeds and pomace. The inclusion of natural pigments in crustaceans and fish diets promotes greater pigmentation [70,73]. The color of fish and shrimp products is an important factor in the selection of products for consumption. Consumers prefer raw shrimps which are grey and lighter in color, and cooked shrimps which are orange in color and more intense [61]. In our study, shrimp fed diets containing pumpkin seeds showed a reddish color after cooking. However, intense orange coloration was more evident in shrimps fed PP diets. Thus, the relationship between total carotenoid content and the increase in the reddish color of shrimp after cooking was established. Our findings agree with those of Fawzy et al. [74], who reported that dietary supplementation with β-carotene for *P. vannamei* shrimp increased redness.

## 5. Conclusions

The inclusion of pumpkin seeds in the diets of shrimp *P. vannamei* resulted in reduced growth parameters and exerted a pro-oxidant effect on shrimp muscle. However, the shrimp body showed improved color after cooking. The inclusion of pumpkin pomace improved the growth parameters, total carotenoid content and shrimp body color. Based on these results, the inclusion of Japanese pumpkin seeds in the diets is not nutritionally viable. We suggest new studies evaluating the inclusion of lower levels of pumpkin seeds in the diet of the shrimp *P. vannamei*. Japanese pumpkin pomace can be included in diets at 100 g·kg^−1^, and further studies are needed to evaluate the possibility of higher levels of inclusion.

## Figures and Tables

**Table 1:** Proximal composition and total carotenoid content of Japanese pumpkin seeds and pomace on a dry matter basis.

Nutrient	Seeds	Pomace
Moisture (%)	9.33 ± 0.45	16.34 ± 1.05
Ash (%)	5.01 ± 0.09	8.15 ± 0.23
Crude protein (%)	21.85 ± 0.38	14.40 ± 0.28
Crude fiber (%)	28.66 ± 0.19	6.71 ± 0.10
Total lipid content (%)	8.32 ± 0.15	2.38 ± 0.05
NFE ^1^ (%)	36.16	68.36
Polyphenols (µg·g^−1^)	685.43 ± 34.92	1650.00 ± 85.85
TCC ^2^ (µg·g^−1^)	0.72 ± 0.03	1.25 ± 0.04

^1^ Nitrogenous-free extract; ^2^ Total carotenoid content.

**Table 2:** Ingredients (g·kg^−1^) and proximal composition (%) of experimental diets with different levels of inclusion of Japanese pumpkin seeds and pomace.

	Diets				
	Control	PS50	PS100	PP50	PP100
Ingredients					
Fish meal ^1^	350	350	350	350	350
Pumpkin seeds ^2^	0	50	100	0	0
Pumpkin pomace ^2^	0	0	0	50	100
Corn starch ^3^	216.5	191.7	166.8	181.1	145.7
Wheat bran ^4^	100	100	100	100	100
Soybean meal ^4^	258.0	236.6	215.3	244.0	230.1
Fish oil ^5^	40.5	36.6	32.8	39.8	39.2
Cholesterol ^6^	5	5	5	5	5
Calcium phosphate ^7^	20	20	20	20	20
Mineral and vitamin premix ^8^	10	10	10	10	10
Proximal composition					
Moisture	5.09 ± 0.13	5.03 ± 0.04	4.85 ± 0.14	4.91 ± 0.09	4.85 ± 0.06
Ash	12.62 ± 0.25	13.08 ± 0.04	12.87 ± 0.15	13.38 ± 0.06	13.91 ± 0.13
Crude protein	34.88 ± 0.45	33.94 ± 0.57	34.18 ± 0.48	34.37 ± 1.13	34.89 ± 1.13
Crude fiber	1.45 ± 0.47	3.14 ± 0.26	5.21 ± 1.93	2.62 ± 0.29	2.44 ± 0.13
Total lipid content	9.40 ± 0.27	9.13 ± 0.30	9.37 ± 0.28	9.71 ± 0.27	9.82 ± 0.34
NFE	41.65	40.71	38.37	39.92	38.94

^1^ Leal Santos, Rio Grande, RS, Brazil; ^2^ Massas Nona Oliva, Vacaria, RS, Brazil; ^3^ Tok, Vogel Alimentos, Canoas, RS, Brazil; ^4^ Sulino, RS, Brazil; ^5^ Campestre^®^, SP, Brazil; ^6^ INLAB, RS, Brazil; ^7^ Dinamica Quimi. Contemp. Ltd.a, RS, Brazil; ^8^ Vitamin A (500.000 UI·kg^−1^), vitamin D3 (250.000 UI·kg^−1^), vitamin E (5.000 mg·kg^−1^), vitamin K3 (500 mg·kg^−1^), vitamin B1 (1.000 mg·kg^−1^), vitamin B2 (1.000 mg·kg^−1^), vitamin B6 (1.000 mg·kg^−1^), vitamin B12 (2.000 mcg·kg^−1^), niacin (2.500 mg·kg^−1^), calcium pantothenate (4.000 mg·kg^−1^), folic acid (500 mg·kg^−1^), biotin (10 mg·kg^−1^), vitamin C (10.000 mg·kg^−1^), choline (100.000 mg·kg^−1^), inositol (1.000 mg·kg^−1^), selenium (30 mg·kg^−1^), iron (5.000 mg·kg^−1^), copper (1.000 mg·kg^−1^), manganese (5.000 mg·kg^−1^), zinc (9.000 mg·kg^−1^), cobalt (50 mg·kg^−1^), and iodine (200 mg·kg^−1^).

**Table 3:** Water physical and chemical parameters measured during a 60-day feeding trial of *Penaeus vannamei* fed diets with different inclusion levels of Japanese pumpkin seeds and pomace.

	Treatments				
Parameters	Control	PS50	PS100	PP50	PP100
D.O. (mg·L^−1^)	6.20 ± 0.38	6.21 ± 0.37	6.20 ± 0.37	6.20 ± 0.38	6.28 ± 0.38
Temperature (°C)	26.7 ± 0.69	26.6 ± 0.68	26.5 ± 0.69	26.7 ± 0.68	26.6 ± 0.69
pH	8.15 ± 0.07	8.17 ± 0.07	8.17 ± 0.07	8.15 ± 0.08	8.11 ± 0.09
Salinity (ppm)	33.0 ± 2.16	33.0 ± 2.16	33 ± 2.16	33.0 ± 2.16	33.0 ± 2.16
TAN (mg·L^−1^)	0.46 ± 0.16	0.42 ± 0.16	0.47 ± 0.17	0.49 ± 0.18	0.50 ± 0.19
NO_2_^−^ (mg·L^−1^)	0.43 ± 0.48	0.50 ± 0.46	0.41 ± 0.50	0.40 ± 0.46	0.39 ± 0.47
NO_3_^−^ (mg·L^−1^)	0.54 ± 0.36	0.55 ± 0.35	0.53 ± 0.38	0.56 ± 0.36	0.55 ± 0.37

No differences were found in the water quality parameters among treatments using one-way analysis of variance (ANOVA). Data are expressed as the mean ± standard deviation of three replicate groups. D.O.: dissolved oxygen. TAN: total ammonia; NO_2_^−^: nitrite; NO_3_^−^: nitrate.

**Table 4:** Growth performance of *Penaeus vannamei* fed diets with different inclusion levels of Japanese pumpkin seeds and pomace.

	Pumpkin Seeds				Pumpkin Pomace			
Parameters	Control	PS50	PS100	*p*-Value	Control	PP50	PP100	*p*-Value
IW (g)	0.59 ± 0.02	0.60 ± 0.02	0.59 ± 0.01	0.212	0.59 ± 0.02	0.58 ± 0.01	0.57 ± 0.01	0.911
FW (g)	4.18 ± 0.11 ^a^	3.89 ± 0.17 ^b^	3.88 ± 0.29 ^b^	0.009	4.18 ± 0.11	4.22 ± 0.26	4.13 ± 0.19	0.657
WG (g)	3.58 ± 0.12 ^a^	3.29 ± 0.15 ^b^	3.28 ± 0.29 ^b^	0.007	3.58 ± 0.12	3.64 ± 0.27	3.56 ± 0.18	0.673
FI (g)	6.94 ± 0.40	6.83 ± 0.66	6.40 ± 0.22	0.054	6.94 ± 0.40	6.62 ± 0.32	6.63 ± 0.27	0.099
PER (%)	1.55 ± 0.25	1.46 ± 0.28	1.56 ± 0.27	0.050	1.55 ± 0.25 ^b^	1.65 ± 0.30 ^ab^	1.67 ± 0.34 ^a^	0.028
FCR (g·g^−1^)	1.93 ± 0.04 ^b^	2.06 ± 0.10 ^a^	1.96 ± 0.11 ^ab^	0.015	1.93 ± 0.04 ^a^	1.82 ± 0.12 ^b^	1.86 ± 0.01 ^ab^	0.020
Survival (%)	92.2 ± 7.08	91.1 ± 4.62	96.6 ± 1.99	0.064	92.2 ± 7.08	93.3 ± 5.08	97.7 ± 2.20	0.831
SGR (%)	3.25 ± 0.08 ^a^	3.09 ± 0.02 ^b^	3.11 ± 0.14 ^b^	0.004	3.25 ± 0.08	3.30 ± 0.13	3.28 ± 0.05	0.528
RWG (%)	605.75 ± 35.69 ^a^	542.49 ± 10.42 ^b^	552.43 ± 54.03 ^ab^	0.003	605.75 ± 35.68	630.52 ± 58.65	617.29 ± 21.45	0.460

Data are expressed as the mean ± standard deviation of three replicate groups. Different letters in the same row indicate statistical differences according to Tukey’s test (*p* < 0.05). IW: initial weight; FW: final weight; WG: weight gain; FI: feed intake; FCR: feed conversion ratio; SGR: specific growth ratio; RWG: relative weight gain.

**Table 5:** Proximal composition of *Penaeus vannamei* (wet weight basis, %) fed diets with different inclusion levels of Japanese pumpkin seeds and pomace.

	Pumpkin Seeds				Pumpkin Pomace			
Composition	Control	PS50	PS100	*p*-Value	Control	PP50	PP100	*p*-Value
Moisture	76.36 ± 0.28	79.35 ± 0.23	79.42 ± 0.28	0.819	79.36 ± 0.28	79.34 ± 0.40	79.09 ± 0.04	0.120
Ash	2.83 ± 0.08 ^b^	2.89 ± 0.06 ^ab^	2.97 ± 0.08 ^a^	0.001	2.83 ± 0.08	2.87 ± 0.06	2.82 ± 0.07	0.300
Crude protein	15.01 ± 0.24	14.64 ± 0.42	14.61 ± 0.85	0.275	15.01 ± 0.24	15.46 ± 0.98	14.94 ± 0.64	0.246
Total lipid	0.58 ± 0.01	0.59 ± 0.02	0.56 ± 0.01	0.074	0.58 ± 0.01	0.60 ± 0.04	0.63 ± 0.08	0.118

Data are expressed as the mean ± standard deviation (*n* = 9). Different letters in the same row indicate statistical differences according to Tukey’s test (*p* < 0.05).

## Data Availability

The data presented in this study are available on request from the corresponding author.
